# Adipose-Derived Mesenchymal Stem Cell Chondrospheroids Cultured in Hypoxia and a 3D Porous Chitosan/Chitin Nanocrystal Scaffold as a Platform for Cartilage Tissue Engineering

**DOI:** 10.3390/ijms21031004

**Published:** 2020-02-03

**Authors:** Veronica Zubillaga, Ana Alonso-Varona, Susana C. M. Fernandes, Asier M. Salaberria, Teodoro Palomares

**Affiliations:** 1 Department of Cell Biology and Histology, Faculty of Medicine and Nursey, University of the Basque Country (UPV/EHU), B Sarriena s/n, 48940 Leioa, Spain; ana.alonsovarona@ehu.eus; 2 Institute of Analytical Sciences and Physico-chemistry for the Environment and Materials, University of Pau and Pays Adour, E2S UPPA, CNRS, 64600 Anglet, France; susana.fernandes@univ-pau.fr; 3 Biorefinery Processes Research Group, Department of Chemical and Environmental Engineering, Polytechnic School, University of the Basque Country (UPV/EHU), Pza. Europa 1, 20018 Donostia-San Sebastian, Spain; asier.martinez@ehu.es; 4 Department of Surgery, Radiology and Physic Medicine, Faculty of Medicine, University of the Basque Country (UPV/EHU), B Sarriena, s/n, 48940 Leioa, Spain

**Keywords:** cartilage tissue engineering, adipose tissue-derived mesenchymal stem cell spheroids, Hypoxia, 3D porous chitosan/chitin nanocrystal scaffold

## Abstract

Articular cartilage degeneration is one of the most common causes of pain and disability in middle-aged and older people. Tissue engineering (TE) has shown great therapeutic promise for this condition. The design of cartilage regeneration constructs must take into account the specific characteristics of the cartilaginous matrix, as well as the avascular nature of cartilage and its cells’ peculiar arrangement in isogenic groups. Keeping these factors in mind, we have designed a 3D porous scaffold based on genipin-crosslinked chitosan/chitin nanocrystals for spheroid chondral differentiation of human adipose tissue-derived mesenchymal stem cells (hASCs) induced in hypoxic conditions. First, we demonstrated that, under low oxygen conditions, the chondrospheroids obtained express cartilage-specific markers including collagen type II (COL2A1) and aggrecan, lacking expression of osteogenic differentiation marker collagen type I (COL1A2). These results were associated with an increased expression of hypoxia-inducible factor 1α, which positively directs COL2A1 and aggrecan expression. Finally, we determined the most suitable chondrogenic differentiation pattern when hASC spheroids were seeded in the 3D porous scaffold under hypoxia and obtained a chondral extracellular matrix with a high sulphated glycosaminoglycan content, which is characteristic of articular cartilage. These findings highlight the potential use of such templates in cartilage tissue engineering.

## 1. Introduction

Articular cartilage is a specialized connective tissue with limited self-repair due to its avascular nature and limited cellularity. The damage and loss of cartilage tissue leads to the progressive remodeling of the subchondral bone leading to osteoarthritis [1], which is the most common and prevalent form of joint disease. The inability of cartilage to repair itself, together with the limited success of current therapies, call for the development of new therapeutic strategies for restoring the functional properties of this type of tissue [2]. Tissue engineering (TE) has undergone a major revolution over the past decade with the development of newly in vitro-formed tissue-material constructs, which exhibit similar biological and mechanical characteristics to those of mature cartilage [3].

The source and availability of cells are key elements to consider in TE constructs. Autologous chondrocytes have been employed in engineered cartilage constructs. Nonetheless, there are specific limitations associated with the use of these cells, namely, cell availability, as well as reduced expansion capacity in vitro and a tendency towards the loss of the differentiated phenotype [4].

Mesenchymal stem cells (MSCs) have been identified as an alternative to autologous chondrocytes as they can be obtained easily, rapidly expand in vitro and are able to differentiate into chondrocytes in the presence of the appropriate growth and transcription factors, including insulin, transferrin, selenium pyruvate and TGF-β [5]. Furthermore, MSCs are able to modulate the immune system, decrease inflammation and participate in wound healing due to their anti-inflammatory, immunomodulatory and paracrine properties [6]. They can be obtained from various types of tissue, with bone marrow and adipose tissue being the main sources used in therapy [7]. Notably, adipose tissue-derived mesenchymal stem cells (ASCs) offer several advantages with respect to bone marrow-derived mesenchymal stem cells, namely: (*i*) higher proliferation capacity, (*ii*) secretion of growth factors related to chondrogenic differentiation (IGF, bFGF and TGF-β1), (*iii*) anti-inflammatory properties, (*iv*) long-term culture stability in the differentiation state, and (*v*) the large numbers of cells that may be harvested from patients with minimally invasive procedures [8,9,10,11,12].

Additionally, human adipose tissue-derived mesenchymal stem cells (hASCs) can be cultured in three-dimensional (3D) structures (spheroids) providing a microenvironment that allows cell–cell and cell-matrix interactions, which are required for maintaining cell viability and promoting chondrogenic differentiation. Compared with monolayer culture, this cellular microenvironment mimics more closely the in vivo chondrification centers that appear during the embryonic development, where mesenchymal chondrocyte precursors tend to form aggregates, thereby intensifying cellular interactions [13,14]. Moreover, cell function and the differentiated phenotype, which is often lost in an in vitro monolayer cell culture, are also enhanced in spheroid culture.

On the other hand, articular cartilage chondrocytes reside in a low oxygen environment due to the absence of blood vessels. Under these conditions, oxygen gradients are between 6% and 10% at the surface of the cartilage and drop to 1% in the deepest layers, indicating that a physiological hypoxic microenvironment is necessary for the maintenance and homeostasis of articular cartilage [15]. In line with this, the microenvironment of hASCs in vivo is also characterized by low oxygen pressure. It has been suggested that hypoxia reduces their potential for osteogenic differentiation [16] while promoting chondral extracellular matrix (ECM) formation [17]. In fact, chondrocyte markers such as SOX9, collagen type II (COL2A1) and aggrecan are positively regulated by hypoxic conditions [18].

By combining the appropriate cell source and culture conditions with highly porous scaffold biomaterials, which act as templates for tissue regeneration, it might be possible to regenerate damaged tissues including articular cartilage. For biomaterials to be effective for such in vivo applications, they must meet some key requirements—namely, chemical versatility, robust mechanical properties, hydrophilicity, high porosity—to ensure cellular penetration, controlled biodegradability and biocompatibility [19,20]. Scaffolds synthesized from natural polymers such as polysaccharides and glycoproteins have most of these properties. Therefore, biopolymers such as chitin (CH) and chitosan (CS) are attractive building blocks and have been widely suggested as good candidates for the development of biocompatible scaffolds [21,22]. CH is a linear polymer composed of N-acetyl-2-amido-2-deoxy-D-glucose units and is commonly found in crustacean and insect exoskeletons and in the cell walls of fungi. At the molecular level, the individual CH chains pile onto each other, forming CH nanofibrils, which can be extracted as CH nanoforms—nanocrystals or nanofibers [23,24,25,26]. Such CH nanoforms have been used as fillers in nanocomposite biomaterials for biomedical applications because of their unique properties, including small size, low density, large surface area, biological activity, and lack of cytotoxicity [21,22].

Furthermore, CS, the main and simplest CH derivative, has also been widely studied for biomedical applications because of its high biodegradability, antimicrobial properties, non-toxicity, biocompatibility, film-forming ability and hydrophilicity [27,28,29]. Despite these properties, CS has certain intrinsic limitations, including poor mechanical properties and uncontrolled swelling in water. To overcome these limitations, and improve the final properties of the 3D scaffold, CH nanoforms have been incorporated into CS matrix [24,30]. CH and CS have already been used in preclinical studies for wound healing [31] and bone regeneration [32]. In our previous work, we demonstrated that CH nanoforms, in particular chitin nanocrystals (CHNCs), provide mechanical and topological cues to support the growth of hASCs in CS-based biomaterials for TE [22].

Herein, to the best of our knowledge for the first time, we describe the development of an in vitro cartilage TE construct that combines hASC spheroids differentiating in a 3D porous scaffold made of genipin-crosslinked chitosan and chitin nanocrystals (CS/CHNCs) under hypoxia. In such low oxygen culture conditions, in the presence of chondrogenic induction medium (CIM) and in the absence of the CS/CHNC biomaterial, chondrospheroids expressed high levels of the chondrogenic differentiation markers COL2A1 and aggrecan. Moreover, these chondrospheroids differentiating inside the CS/CHNC biomaterial maintained a compacted structure resembling the chondrification centers present during embryonic development and resulted in an increase in ECM deposition, in terms of sulphated glucosaminoglycan (sGAG; the main component of proteoglycans) production. The combination of the aforementioned culture conditions allowed us to obtain a cartilage TE construct with optimal chondrogenic differentiation features.

## 2. Results

### 2.1. Influence of Hypoxia on hASC Chondrospheroid Generation

hASC spheroids were cultured in CIM for chondrospheroid generation, both in normoxic and hypoxic conditions for 7, 14 and 21 days. We observed that both types of spheroid displayed compact rounded cell aggregation that maintained their physical structure for the length of the experiment. In order to assess the suitability of this hASC 3D culture model, we undertook cell viability studies and spheroid volume quantification.

**Figure 1:**
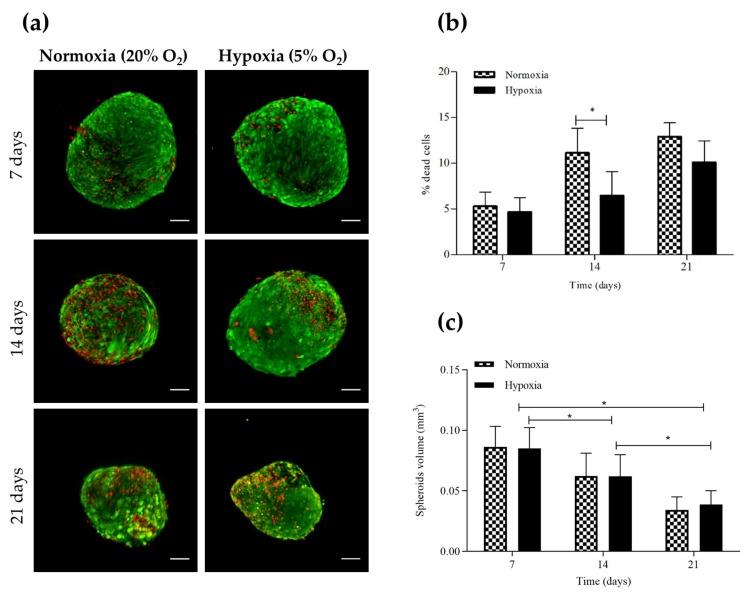
Chondrospheroid characterization in normoxic or hypoxic conditions at 7, 14 and 21 days. (**a**) Images of confocal microscopy showing chondospheroid cell viability assessed using Live/Dead assay (*n* = 4–8 per time and culture condition); (**b**) Percentage of dead cells in chondrospheroids cultured in normoxia or hypoxia at 7, 14 and 21 days (*n* = 4–8 per time and culture condition); (**c**) Spheroid volume quantification (*n* = 50 per time and culture condition). The scale bar represents 100 µm. Two-way ANOVA and Bonferroni correction was performed for the statistical analysis (* *p* < 0.05).

The assessment of cell viability (using a Live/Dead assay) showed high cell viability (cells stained with green fluorescence) on the surface of chondrospheroids (Figure 1a). Specifically, at 7 days of culture, the percentage of dead cells (stained with red fluorescence) was 5.32% ± 1.54% and 4.7% ± 1.6% in normoxia and hypoxia, respectively. At Days 14 and 21, the proportion of dead cells was higher in chondrospheroids cultured in normoxia than those cultured in hypoxia (11.2% ± 2.7% and 6.48% ± 2.56% at 14 days, and 12.9% ± 1.5% and 10.10% ± 2.36% of dead cells at 21 days), these differences being statistically significant at 14 days of culture (Figure 1b).

The chondrospheroid volume quantification can be used as an indicator of cell proliferation, matrix deposition and intercellular space volume [33]. Regarding the influence of oxygen concentration on the chondrospheroid volume, no significant differences were found between the two groups (volume of 0.086 ± 0.017, 0.0623 ± 0.019, 0.034 ± 0.01 mm^3^ in normoxia, and 0.085 ± 0.02, 0.062 ± 0.017 and 0.038 ± 0.01 mm^3^ in hypoxia, at 7, 14 and 21 days, respectively; Figure 1c). Moreover, from 7 to 21 days of culture there was a similar shrinking trend (near 60% reduction) in the volume of both types of chondrospheroid, which was statistically significant between 7 and 14, 7 and 21 and 14 and 21 days.

### 2.2. Chondrogenic Differentiation of hASC Spheroids in Hypoxia 

The differentiation level of the chondrospheroids was assessed by histological staining and Western blot analysis.

**Figure 2:**
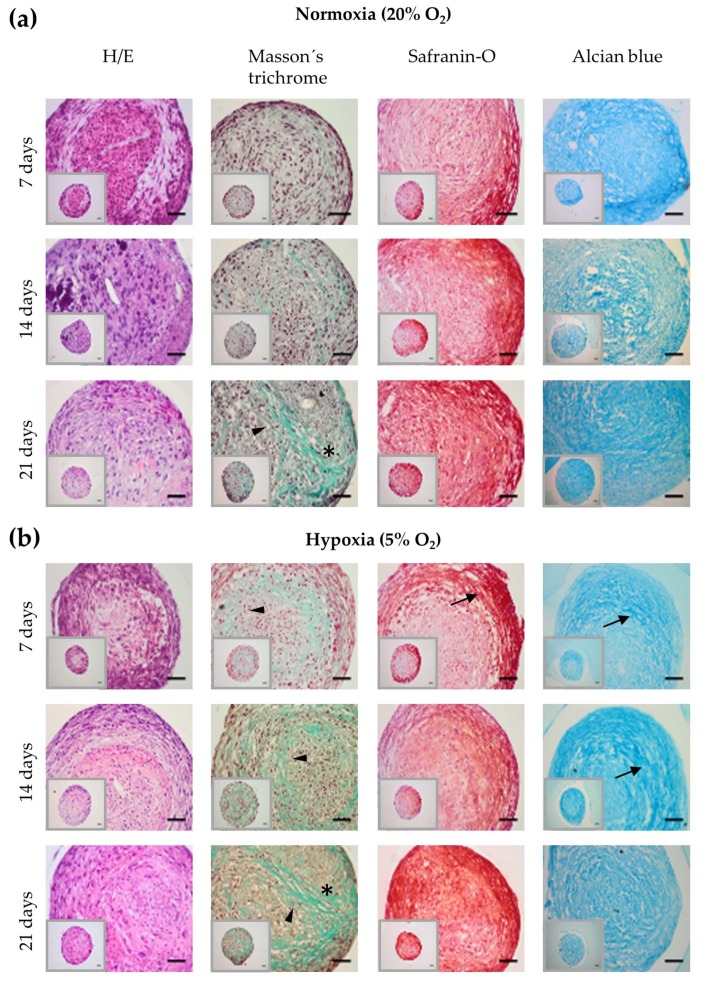
Bright field images of the histological sections obtained from chondrospheroids cultured at 7, 14 and 21 days under (**a**) normoxic and (**b**) hypoxic conditions stained with H/E, Masson’s trichrome, safranin-O and alcian blue (*n* = 10 spheroids per time and culture condition). Central region of the spheroids (Masson’s trichrome): arrowheads mark collagen distribution and asterisks (*) mark chondrocyte-like cells surrounded by collagen fibers they had synthesized and secreted. Periphery of the spheroids (safranin-O and alcian blue): arrows mark sGAG distribution. The scale bar represents 50 µm.

The histological evaluation of the chondrospheroids showed ECM deposition in terms of collagen and sGAGs synthesis in both normoxic and hypoxic conditions, as can be observed in the micrographs of the histological sections stained with Masson’s trichrome, alcian blue and safranine-O (Figure 2). In the case of the collagen synthesis (Masson’s trichrome staining), we observed that most fibers appeared at the center of the spheroid at early stages of chondrogenic differentiation (7 and 14 days); however, as the chondrogenesis progressed, thick and heavily stained collagen fibers were observed across the center and at the periphery of the spheroids. At Day 21, high magnification images showed cells surrounded by the collagen fibrils they had secreted during their chondrogenic differentiation. Regarding sGAGS (safranin-O and alcian blue staining), micrographs show intense ring-shaped sGAG deposition from the periphery (proliferating zone) towards the core of the spheroid. In the same way as with collagen, the safranin-O staining revealed high sGAG deposition on Day 21 (Figure 2a,b).

On the other hand, there were differences in the dynamic process of chondrogenic differentiation between normoxic and hypoxic conditions (Figure 2b). Specifically, after 7 days of culture, Masson’s trichrome and alcian blue staining showed higher chondrogenic differentiation in the chondrospheroids cultured under hypoxia than those maintained in normoxia (Figure 2a). These differences were not observed at Day 21, by which point the collagen and sGAG deposition were similar in the two types of spheroids.

**Figure 3:**
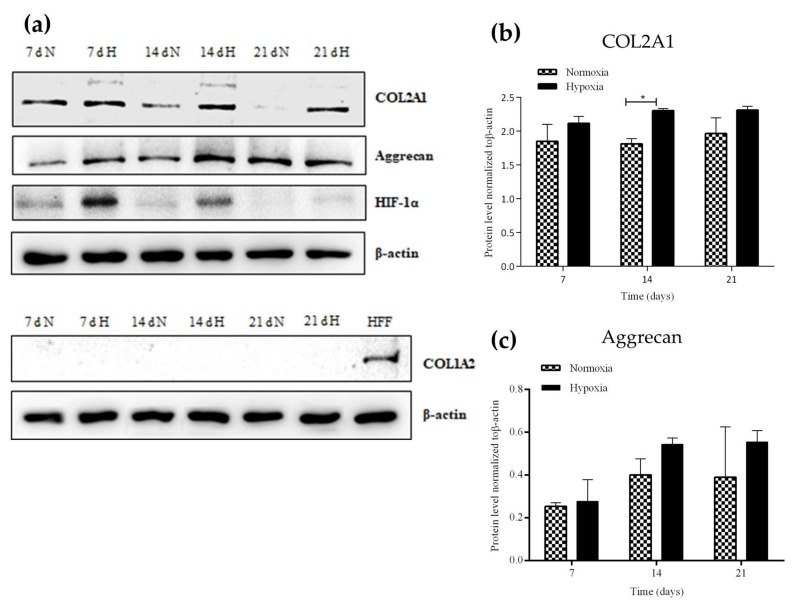
Analysis of (**a**) COL2A1, aggrecan, HIF-1α and COL1A2 protein expression in the chondrospheroids by Western blot (n_spheroids_ = 10). As an internal positive control for COL1A2 expression, a human foreskin fibroblast (HFF) cell line was used. (**b**) COL2A1 and (**c**) aggrecan quantifications were normalized to β-actin. N: normoxia (20% O_2_); H: hypoxia (5% O_2_); d: days. Two-way ANOVA and Bonferroni correction was performed for the statistical analysis (* *p* < 0.05).

To add further support to the observations found by specific histological staining, the differentiation level was also assessed through the analysis of COL2A1, aggrecan and COL1A2 expression by Western blot. The results reveal that both COL2A1 and aggrecan proteins were expressed in the chondrospheroids, regardless of whether they had been cultured under normoxia or hypoxia (Figure 3a). In accordance with our earlier results, however, greater chondrogenic differentiation was observed at 7, 14 and 21 days of culture under hypoxia, as shown by the Western blot quantification (Figure 3b,c), which is statistically significant at 14 days for COL2A1 expression (Figure 3b)**.** Furthermore, in these chondrospheroids, the COL2A1 antibody detected a prominent band with a molecular mass of ~130 kDa and a second band at ~140 kDa, at 7 and 14 days of culture in hypoxia, corresponding to the two COL2A1 isoforms. In the case of collagen type I (COL1A2)—present in skin, bone and fibrocartilage, but not in hyaline cartilage—no expression was detected in any chondrospheroids, indicating only cartilage-like features inside the spheroids.

In addition to that of the chondrogenic differentiation markers, the expression of hypoxia inducible factor (HIF-1α) was also studied. This factor positively regulates both COL2A1 and aggrecan expression and is expressed by cells in response to low oxygen levels [34]. Our results reveal HIF-1α expression in chondrospheroids at 7 and 14 days of culture both under normoxic and hypoxic conditions, this expression being significantly higher in chondrospheroids cultured under hypoxia. This high expression of HIF-1α might be responsible for the elevated COL2A1 and aggrecan expression at these times.

### 2.3. hASC Spheroid Adhesion, Viability and Chondrogenic Differentiation in the 3D Porous CS/CHNC Scaffold under Hypoxia

**Figure 4:**
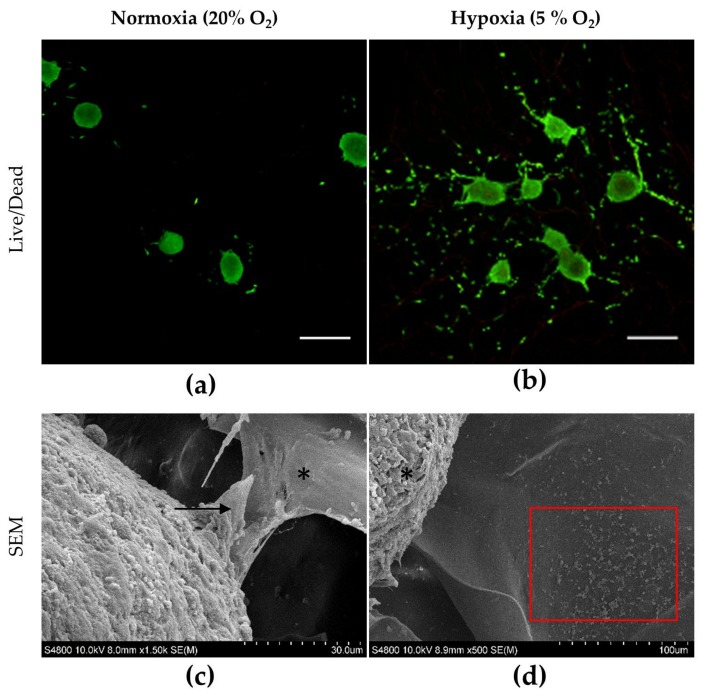
Viability (Live/Dead assay) of adhered hASC spheroids cultured in CM for 7 days in contact with the 3D porous CS/CHNC scaffold studied under (**a**) normoxia and (**b**) hypoxia. Images of hASC spheroids adhered (SEM) to the aforementioned biomaterial at 7 days under (**c**) normoxia and (**d**) hypoxia. The asterisks (*) indicate the scaffold and the arrows the interaction between the cells forming the spheroids and the biomaterial. The red square represents individual cells that have egressed from the spheroid under hypoxia conditions. Scale bar in the fluorescence images represents 500 µm.

We first analyzed the viability and adhesion of hASC spheroids (maintained in CM) to the 3D porous CS/CHNC scaffold at 7 days, under normoxic and hypoxic conditions. At the time of seeding, the diameter of the hASC spheroids was 305 ± 22 µm, a size that allows them to penetrate into the pores of the biomaterial. As observed in the Live/Dead images (Figure 4a,b), under both culture conditions, hASC spheroids showed high viability and adhered well to the exposed surface of the biomaterial. Nonetheless, under hypoxia, a significantly larger number of cells egressed from the spheroids, colonizing a greater extent of the biomaterial surface (Figure 4b), compared to the case of the hASC spheroids cultured under normoxia (Figure 4a). Scanning electron microscope (SEM) images show in detail that spheroids cultured under normoxia adhere to the material, maintaining their typical compact structure (Figure 4c). In contrast, spheroids cultured in hypoxia exhibited egressed cells with long cytoplasmic projections that facilitated cell adhesion to the 3D porous CS/CHNC scaffolds (Figure 4d). These data indicate that the CS/CHNC biomaterial provided an adequate substrate for hASC spheroid adhesion and that the hypoxic conditions allowed cells to cover a high proportion of the surface of the biomaterial.

**Figure 5:**
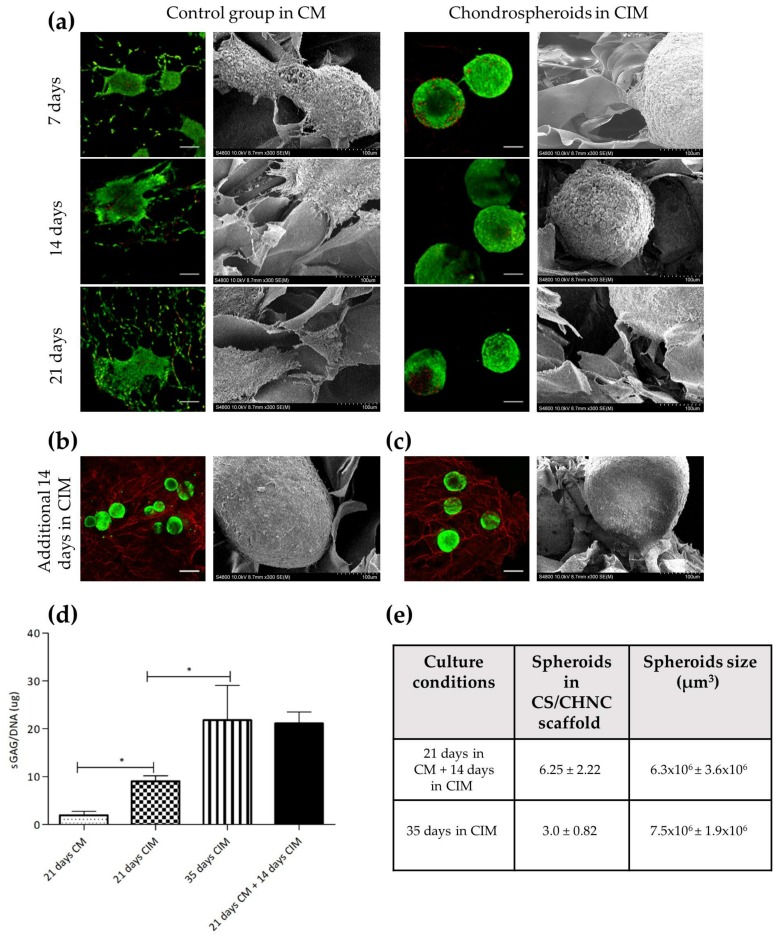
Establishment of the cartilage tissue engineering (TE) construct based on hASC spheroids adhered to the 3D porous CS/CHNC scaffold in hypoxic conditions. Viability (Live/Dead assay) and adhesion (SEM) of the (**a**) control group spheroids and chondrospheroids at 7, 14 and 21 days, (**b**) hASC spheroids cultured at 21 days in CM plus an additional 14 days in CIM and (**c**) hASC spheroids cultured for 35 in CIM. (**d**) Quantification of the ECM deposition, in terms of sGAG/DNA (µg), from spheroids cultured 21 days in CM, 21 days in CIM, 21 days in CM plus an additional 14 days in CIM and 35 days in CIM. (**e**) Number of spheroids distributed through the scaffold and spheroid size. The scale bar represents (**a**) 200 µm and (**b**) 500 µm in the fluorescence images ×300 magnification in the SEM images. Three constructs containing 20 spheroids for each time and condition were used. Data shown are mean ± SD (* *p* < 0.05; one-way ANOVA).

Considering these results, together with the fact that the in vivo environment of the hASCs and articular cartilage is hypoxic, further studies with longer culture times to assess cell viability, adhesion to the material and chondrogenic differentiation were carried out only under low oxygen conditions. In this hypoxic microenvironment, and in concordance with the results obtained previously and shown in Figure 4b, in the case of hASC spheroids cultured in CM, cells egressed from the spheroid that progressively colonized the exposed surface of the biomaterial from 7 to 21 days. Similarly, SEM images show cytoplasmic projections interacting with the surrounding 3D porous CS/CHNC scaffold. In contrast, spheroids cultured in CIM maintained the typical compact physical structure of chondrospheroids for the length of the experiment (21 days; Figure 5). These results suggest that the 3D CS/CHNC scaffold is suitable for supporting hASC spheroids differentiating as chondrification centers.

Secondly, in order to determine the most suitable differentiation pattern, we designed an experimental protocol in which hASC spheroids were seeded in the 3D porous CS/CHNC scaffold and cultured under low oxygen tension in (*i*) CM (21 days) + CIM (14 days) or (*ii)* in CIM (35 days). Although hASC spheroids cultured in CM lost their compact structure due to the egression of cells, when CM was replaced by CIM, the cells tended to aggregate again, forming compact chondrospheroids that resembled the chondrification centers present during cartilage development (Figure 5b). In contrast, samples cultured for 35 days in CIM maintained the compact structure throughout the incubation period (Figure 5c). Interestingly, compared to the samples cultured for 35 days in CIM, those cultured for 21 days in CM and an additional 14 days in CIM contained a larger number of spheroids distributed homogeneously through the CS/CHNC scaffold (3.0 ± 0.82 vs. 6.25 ± 2.22 spheroids per microscopical field of the scaffold, respectively; *p* < 0.05) and smaller spheroid size (0.075 ± 0.19 vs. 0.063 ± 0.036 mm^3^, respectively, *p* = 0.83). We then proceeded to quantify the sGAG content in the spheroids differentiated in the 3D CS/CHNC scaffold. The ECM of hyaline cartilage consists of a fibrillar collagen network with entrapped sGAG molecules, and hence, sGAG has also been considered a marker of chondrogenic differentiation. Our results reveal that control group hASC spheroids produced a significantly lower amount of sGAG than chondrospheroids cultured in CIM for 21 days (1.960 ± 0.80 and 9.1 ± 1.13 µg sGAG/DNA, respectively). On the other hand, chondrospheroids from both 35-day culture groups (21 days in CM plus an additional 14 days in CIM, or 35 days in CIM) reached similar chondrogenic differentiation levels (producing 21.10 ± 2.40 and 21.77 ± 7.28 µg sGAG/DNA, respectively; Figure 5d).

## 3. Discussion

Articular cartilage repair is one of the main targets for TE. In this work, as key elements for the design of a TE construct, we used hASC spheroids as the cell source, a biomaterial based on CS and CHNCs obtained from renewable marine resources, a specific CIM and a low oxygen atmosphere. As demonstrated previously by other authors, hASC spheroids provide controlled spatial organization and enhanced cell-cell interactions, mimicking crucial events occurring during cartilage morphogenesis [35]. The hanging-drop culture technique employed in this work for the formation of spheroids has been previously used for generating 3D chondral structures [33] and small pieces of cartilage [36]. Biomaterials can promote cartilage formation facilitating this condensation process, while providing an optimal system for the delivery of spheroids to damaged tissue [37]. Additionally, hypoxia seems to be a key factor in the design of this construct, due to the avascular nature of articular cartilage, providing a more favorable microenvironment for chondrogenesis [38]. In fact, our chondrospheroid viability studies revealed larger numbers of dead cells in chondrospheroids cultured in normoxia than in those cultured in hypoxia. These results are in line with those of other authors, who have shown that hASC populations cultured in hypoxia exhibited better growth and survival [39,40,41], with few apoptotic events [17].

Regarding the influence of oxygen concentration on the volume of chondrospheroids, we did not find differences between spheroids cultured in normoxia and hypoxia. Specifically, a similar volume reduction was observed during chondrospheroid generation in both cases. Our findings correlate with those of another study which found a similar sized decrease due to the intense cell-cell contact compaction in the spheroids and the retracting forces exerted by the cells on the ECM fibrils [42]. Other authors have described the specific microenvironment and geometry inside such spheroids, which comprises proliferating, quiescent and necrotic regions. Thus, different mass transport rates for nutrients, O_2_, adenosine triphosphate, waste, CO_2_ and lactase are present in the multilayer structure inside these spheroids [43]. We have also observed a regional distribution pattern regarding ECM protein synthesis, sGAG and collagen fibers being located at the periphery and in the center of the chondrospheroids, respectively. Similar results have previously been reported in MSC spheroids during chondrogenic differentiation [13,44,45].

In relation to chondrogenic differentiation markers, some authors have described the expression of COL2A1 and aggrecan in human adult MSCs differentiated in pellet culture [46]. Our assessment of these markers by Western blot revealed the expression of both proteins in spheroids cultured under normoxia and hypoxia, but greater chondrogenic differentiation was observed in the case of chondrospheroids cultured under hypoxia. Moreover, we found a second band at a higher molecular size only in the case of COLA21 expression in chondrospheroids cultured under low oxygen conditions. This result could be explained by an alternative splicing in pre-mRNA of the type II procollagen gene in exon 2 (COL2A1 in humans), during cartilage development, this resulting in two isoforms of type II procollagen. Type IIA isoforms contain exon 2, while type IIB isoforms lack this exon. This event is developmentally regulated during chondrogenesis, where chondroprogenitor cells predominantly express IIA isoforms, while differentiated chondrocytes produce mainly IIB [47,48]. Considering this feature, our results suggest the presence of cell populations with different chondrogenic differentiation levels within the spheroids.

In addition to that of the chondrogenic differentiation markers (COL2A1 and aggrecan), the expression of HIF-1α was also studied. HIF-1α is expressed in cells as a response to low oxygen levels and positively regulates both COL2A1 and aggrecan expression [34]. Our data revealed a significantly higher expression of HIF-1α at 7 and 14 days in chondrospheroids cultured under hypoxia than in those cultured under normoxia, which explains the higher COL2A1 and aggrecan expression at the same time points. These findings are in line with those obtained by other authors, in which hypoxic conditions were associated with the increased expression of cartilage-related biomarkers and biosynthesis of a glycosaminoglycan-positive matrix in 3D structures [15,49] and the maintenance of the chondrogenic phenotype in chondrocytes [50].

Hypoxia, besides promoting chondrogenic differentiation of hASCs [16], also downregulates osteogenesis [51] and inhibits endochondral ossification with low expression in Runx2 and COL10A1 [52]. Notably, Xu et al. [53] found that hypoxic preconditioning enhanced the chondrogenic differentiation and decreased osteogenic differentiation of murine ASCs. Our results indicate that the osteogenic marker COL1A2 is not expressed at all in chondrospheroids, regardless of the culture conditions. It should be considered that in chondrospheroids maintained in normoxia, the 3D structure itself leads to a reduction in the local level of oxygen, which explains the inhibition of the expression of COL1A2. In the case of chondrospheroids cultured in hypoxia, the 3D structure, together with these low oxygen culture conditions, led to even lower local oxygen tension throughout the spheroids [13], increasing the chondrogenic differentiation level.

As we have mentioned before, another key element of cartilage TE constructs is the biomaterial that will host the cells and promote cartilage formation. Herein, we proposed a 3D porous scaffold biomaterial developed in our recent work [22] based on genipin-crosslinking CS and CHNCs. The ideal scaffold should resemble the molecules present in cartilage ECM—mainly collagen and GAGs—and the physicochemical properties of CS and CH have been shown to play a significant role in cartilage TE due to their structural similarity with the GAGs present in the ECM of cartilage tissue [54,55]. Moreover, we recently demonstrated that the incorporation of rigid substrates like CHNCs into a genipin-crosslinked CS matrix improves the mechanical properties and stiffness (Young’s modulus [0.4 ± 0.05 MPa], compression strength [0.37 ± 0.05 MPa], and strain recovery [27.0 ± 2.8%]), swelling properties and cell compatibility of the biomaterial. Controlling the physicochemical and mechanical properties of the 3D porous scaffold biomaterial, we can improve cell adhesion and proliferation. Furthermore, this 3D porous CS/CHNC scaffold showed a porosity of around 95% ± 3% and pore size from 250 to 500 μm, which are important requirements for spheroid retention, adhesion and chondral differentiation [22]. In relation to this, in the present work, we observed that hASC spheroids adhered well to the 3D porous CS/CHNC scaffold.

Another objective of this work was to explore the best differentiation pattern to achieve optimal coverage of the 3D porous CS/CHNC scaffold. We found that the biomaterial in which hASC spheroids were cultured for 21 days in CM and an additional 14 days CIM contained larger numbers of spheroids than the samples cultured for 35 days in CIM. This could be explained by cells from spheroids cultured initially in CM leaving them and spreading more widely through the 3D porous CS/CHNC scaffold. Specifically, when the CIM was added, more chondrospheroids where formed across the exposed surface of the biomaterial. This may be important for achieving a homogeneous arrangement of chondrospheroids through the 3D porous scaffold. The level of differentiation of these chondrospheroids was evaluated by the quantification of ECM synthesis. The ECM of hyaline cartilage consists of a fibrillar collagen network with entrapped sGAG molecules, and hence, sGAG has also been considered a chondrogenic differentiation marker. Good differentiation capabilities have previously been described in hASC spheroids seeded in poly L-glutamic acid/CS [56] and CS/hyaluronan scaffolds, in terms of cartilage specific sGAG gene expression [57]. Our results from the sGAG quantification suggest that (*i*) the presence of CIM is needed to synthesize sGAG and (*ii*) a longer exposure time in CIM (35 days compared to 21 days) produces greater sGAG deposition. Nonetheless, we obtained the same level of sGAG synthesis when the spheroids were cultured initially in CM for 21 days and only an additional period of 14 days in CIM. This chondrogenic differentiation pattern allows a more homogenous chondrospheroid distribution through the 3D porous CS/CHNC scaffold with significant sGAG synthesis, comparable to that obtained in the samples cultured for 35 days in CIM.

## 4. Materials and Methods

### 4.1. hASC Spheroids Formation and Chondrogenic Differentiation

hASCs, kindly supplied by Histocell SL. (Bizkaia Science and Technology Park, Zamudio, Spain), were maintained in control medium (CM) containing Dulbecco´s Modified Eagle Medium (DMEM)-Glutamax^TM^ (Gibco, Paisley, UK), supplemented with 1% penicillin-streptomycin (Lonza, Verviers, Belgium) and 10% fetal bovine serum (Biochrom AG, Berlin, Germany).

In order to obtain hASC spheroids, the hanging-drop technique was used. This method allows spontaneous cell aggregation by culturing cell suspensions on the inner side of tissue culture dishes. Several different cell densities were studied; namely, 5 × 10^3^, 1 × 10^4^ and 1.5 × 10^4^ cells per spheroid. As reported by other authors [58], 1.5 × 10^4^ exhibited the highest metabolic activity, the lowest level of apoptosis and the most uniform proliferating cell distribution. In our case, this cell density also achieved the best level of chondrogenic differentiation. Briefly, drops of 30 µL of CM containing 1.5 × 10^4^ cells were seeded on the cover of a plastic petri dish (J.D. Catalán, Spain) and allowed aggregate over 48 h at 37 °C in a humidified atmosphere containing 5% CO_2_. After this time, two spheroids per well were transferred into a 24-well ultralow attachment culture plate (Corning, NY, USA). In the case of the control group, 200 µL of the CM were added to each well. For chondrospheroids (the chondrogenic differentiation group), the samples were washed in sterile phosphate buffered saline (PBS 1×; Sigma-Aldrich, Saint Louis, USA) and 200 µL of the CIM (STEMPRO^®^ Chrondrogenesis Differentiation Kit; Gibco, Paisley, UK) supplemented with 1% penicillin-streptomycin. Both types of spheroid were maintained for 7, 14 and 21 days under either normoxic (20% O_2_) or hypoxic (5% O_2_) conditions. The media were replaced twice a week.

### 4.2. Chondrospheroid Volume Quantification

Chondrospheroids cultured in normoxia and hypoxia were imaged on days 7, 14 and 21 using IC Capture 2.3 under a phase-contrast microscope (Nikon Eclipse TS100, Tokyo, Japan). Spheroid diameters were measured using a computer-based image analysis system (Image J, NIH, USA) and the volume was calculated assuming perfect spheres (n_spheroid_ = 50 per time and culture condition).

### 4.3. Chondrospheroid Viability

In order to evaluate chondrospheroid viability, a Live/Dead assay was performed (n_spheroids_ = 4–8). Chondrospheroids cultured for 7, 14 and 21 days under normoxia and hypoxia were washed in 1× PBS and incubated with 4 µM of calcein-AM (Sigma-Aldrich, St. Louis, USA) and 5 µM propidium iodide (Molecular Probes, Eugene) in PBS for 15 min at 37 °C. The samples were observed under a confocal microscope (Olympus LV500, Japan) to visualize viable (green fluorescence; λ_ex_-λ_em_ = 490–515 nm) and dead (red fluorescence; λ_ex_-λ_em_ = 490–630 nm) cells. The percentage of dead cells was quantified using the aforementioned computer-based image analysis system (Image J). The experiment was carried out in triplicate, measuring one or two spheroids per well.

### 4.4. Histological Analysis

For the histological analysis of the chondrogenic markers, chondrospheroids cultured under both normoxia and hypoxia (n_spheroids_ = 10 chondrospheroids for each oxygen condition) were harvested at 7, 14 and 21 days, washed in 1× PBS and fixed in paraformaldehyde 4% (PanReac Applichem, Barcelona, Spain) for 20 min at room temperature. Fixed chondrospheroids were washed in 1× PBS, embedded in 1% agarose (Conda, Madrid, Spain) in PBS to facilitate the handling and fixed again in paraformaldehyde 4% for 1 h at 4 °C. Then, samples were dehydrated with serial concentrations of ethanol (50–100%), immersed twice in methyl benzoate (VWR Chemicals, Leuven, Belgium), citrosol (PanReac AppliChem, Barcelona, Spain) and embedded in paraffin wax (Fibrowax^TM^ VWR Chemicals, Leuven, Belgium). Subsequently, 5-µm thick histological sections, taken from the central zone of the spheroid, were immersed in citrosol and rehydrated in a series of decreasing ethanol concentrations followed by washing in tap water for 5 min.

Histological sections were stained with Masson´s trichrome for collagen detection and with safranin-O (0.1% *w/v*) and alcian blue (1% *w/v*; both from Sigma-Aldrich, St. Louis, U.S), for sGAG detection (sGAG staining orange to red and blue, respectively). As a histological reference, hematoxylin and eosin (H&E) stains (both from Thermo Fisher Scientific, Cheshire, UK) were also used. After the staining, all the sections were dehydrated with ethanol followed by immersion in citrosol for 5 min three times, and then mounted with a coverslip using DPX mountant (Sigma-Aldrich, St. Louis, USA). The samples were examined under a bright field microscope (Olympus BX50) and images were taken using Cell^A^ software (Olympus Soft Imaging Solutions). The experiment was carried out in triplicate, with five replicates containing two spheroids per well.

### 4.5. Western Blot

Chondrospheroids cultured under normoxia and hypoxia were collected at 7, 14 and 21 days (n_spheroids_ = 10) and washed in 1× PBS. After that, 200 µL of Laemmli buffer (Sigma-Aldrich, St. Louis, USA) were added and samples were lysed by sonicating with three 30-s pulses (Sonopuls, Berlin, Germany). The protein concentration was determined using the method for protein assay based on trichloroacetic acid, as described by other authors [59].

Equal amounts of protein (10 µg) were separated by 10% SDS-PAGE and transferred onto nitrocellulose membranes (Amersham^TM^ Protram^TM^, GE Healthcare, Life Science, Germany) for 3 h at 380 mA. The membranes were blocked in 5% skimmed milk for 1 h at room temperature and subsequently incubated with anti-mouse COL2A1, aggrecan (4F4) (both at 1:200), HIF-1α (BS Bioscience U.S; 1:250) and anti-rabbit COL1A2 (Genetex, Barcelona, Spain, 1:1000) and β-actin (EDM Millipore, Temecula, U.S 1:5000) primary antibodies at 4 °C overnight. Then, the membranes were incubated with goat anti-mouse IgG-HRP (Santa Cruz Biotechnology, Dallas, USA; 1:1000) and donkey anti-rabbit IgG HRP secondary antibodies (EDM Millipore, Temecula, USA; 1:1000) for 1 h at room temperature. The membranes were visualized using Luminata^TM^ Crescendo Western HRP Substrate (EDM Millipore, Temecula, USA), and images were acquired with the G:BOX Chemi HR16 gel documentation system (Syngene, Frederick, USA). The relative protein expression levels were normalized to that of their corresponding loading control β-actin. Human foreskin fibroblasts (HFF) expressing COL1A2 were used as an internal control for the COL1A2 Western blot. The experiment was carried out in triplicate, using five replicates containing two spheroids per well. The quantification was made using ImageJ software.

### 4.6. Synthesis of the 3D Porous Genipin-Crosslinked CS/CHNC Scaffold 

3D porous genipin-crosslinked CS/CHNC scaffolds were prepared by freeze drying following the approach described in our previous work [22]. Briefly, 1.0% w/v solution was first prepared by dissolving CS (DDA = 98% and 500,000 g mol^−1^ extracted from squid and processed to medical grade, Mahtani Chitosan Pvt. Ltd., India) in 1.0% *v/v* aqueous acetic acid (99%, ReagentPlus) under vigorous stirring at 25 ± 1 °C for 48 h. After dissolution, 0.5% *w/w* of genipin (≥98%, Sigma-Aldrich Europe), proportionate to the dry weight of CS, was added and stirred at 25 ± 1 °C for 15 min. Then, never-dried CHNCs (prepared in-house [23] from CH powder extracted from lobster exoskeletal waste kindly supplied by Antarctic Seafood S.A., Chile), with a CS:CHNC weight ratio of 1:0.5, were added and dispersed using an Ultra-Turrax machine at 20,000 rpm for 30 min. Finally, the 3D porous scaffolds were prepared by transferring the suspension into cylindrical containers followed by storage at 40 °C for 24 h to create the cross-linking reaction and subsequently frozen at −20 °C for 72 h. Thereafter, the samples were lyophilized using a Martin Christ Alpha 1-4 LDplus freeze dryer (Germany) with a condenser temperature of −55 °C for 4 days. The resulting samples were trimmed to 3D porous scaffolds with an apparent size of 4.5 cm height and 3.0 cm diameter. The CS/CHNC 3D porous scaffold showed a porosity of around 95% ± 3%, measured by the principle of liquid displacement [22]. Forty pores of the cross-section were measured and quantified from the SEM images to determine the porous size of the scaffold that showed values from 250 to 500 μm [22]. The mechanical properties were evaluated by compression testing and showed a Young’s modulus of 0.37 MPa and strain recovery of 27%. The samples were kept in a conditioning cabinet at 50% relative humidity (RH) and 25 °C to ensure the stabilization of their water content.

### 4.7. hASC Spheroids Adhesion and Viability in the 3D Porous CS/CHNC Scaffold

The 3D porous CS/CHNC scaffolds (0.3 cm^3^) were sterilized in 70% ethanol for 2 h, washed three times in sterile 1× PBS, placed in a 24-well ultra-low attachment plate (Corning NY, USA) and pre-wetted in CM for 24 h at 37 °C. Drops of 15 µL of CM containing 20 spheroids were seeded onto scaffolds and allowed to adhere for 5 h at 37 °C in a humidified atmosphere containing 5% CO_2_ under normoxia. After this time, hASC spheroids were either kept under normoxia or transferred to hypoxia.

In order to decide the best oxygen concentration conditions for the culture, the samples were first cultured for 7 days in CM, either under normoxia or hypoxia. Based on the results obtained, the samples were subsequently cultured under hypoxia and the following conditions: (*i*) 7, 14 and 21 days in CM, (*ii*) 7, 14 and 21 days in CIM, (*iii*) 21 days in CM followed by 14 days in CIM, and (*iv*) 35 days in CIM.

Cell adhesion and viability of adherent spheroids on the 3D porous CS/CHNC scaffolds were evaluated by Live/Dead assay (as described previously) and scanning electron microscopy (SEM). For SEM analysis, all samples were rinsed three times in Sorensen buffer (PanReac AppliChem, Barcelona, Spain), fixed using 2% glutaraldehyde (PanReac AppliChem, Barcelona, Spain) for 2 h, washed in buffer, dehydrated using a series of gradient ethanol solutions and dried in hexamethyldisilazane (Sigma-Aldrich, Saint Louis, USA)) for 10 min. The samples were sputtered with a thin layer of gold under an argon atmosphere and observed using a Hitachi S-4800 SEM with an accelerating voltage of 10 kV. The experiment was carried out in triplicate.

### 4.8. Chondrogenic Differentiation in the 3D Porous CS/CHNC Scaffolds

In order to determine the chondrogenic differentiation of spheroids (cultured in CM or CIM) seeded onto the 3D porous CS/CHNC scaffolds, the sGAG content was quantified at 21 and 35 days of culture. The samples were harvested, washed with 1× PBS and digested with 125 µg/mL papain extraction reagent in 0.2 M sodium phosphate buffer, 400 mg sodium acetate, 200 mg ethylenediaminetetraacetic acid and 40 mg cysteine HCl for 3 h at 65 °C. The digested extracts were centrifuged at 10,000× *g* for 10 min and the supernatants were collected. The sGAG content was determined with a 1,9-dimethylmethylene blue colorimetric assay (Blyscan sGAG assay Kit, Biocolor, Northern Ireland, UK) according to the manufacturer´s protocols and the absorbance of each solution was measured at 655 nm (Synergy HT spectrophotometer, BioTek, USA). The amount of sGAG was normalized to the DNA content for each sample (DNA Quantitation Kit; Sigma-Aldrich, St. Louis, USA).

### 4.9. Statistical Analysis

Statistical analysis was performed using one-way and two-way analysis of variance (ANOVA) followed by a post hoc Bonferroni correction and Student’s t test. The results are expressed as mean ± SD and values of *p* < 0.05 were considered to be statistically significant.

## 5. Conclusions

In conclusion, we used a cartilage TE construct based on 3D porous genipin-crosslinked CS/CHNC scaffold and hASC spheroids maintained in hypoxic conditions as a platform for articular cartilage regeneration. The spheroids, when cultured in the appropriate chondrogenic induction media, showed a high level of COL2A1 and aggrecan matrix deposition, while lacking COL1A2 expression, and demonstrated adequate chondrogenic differentiation. Moreover, under low oxygen tension and with the appropriate chondrogenic induction media administration pattern, the differentiation of the hASC spheroids in the 3D porous CS/CHNC scaffold allowed the production of a chondral ECM with significant sGAG synthesis. The TE construct mimics the in vivo cartilage environment during the embryonic development, and this entails a further step in cartilage TE, with potential applications for articular cartilage regeneration.
